# Metagenomic Analysis Revealed the Changes in Antibiotic Resistance Genes and Heavy Metal Resistance Genes in Phosphate Tailings Compost

**DOI:** 10.3390/microorganisms13040768

**Published:** 2025-03-28

**Authors:** Chunqiao Xiao, Kai Wan, Yan Chen, Yongtong Jin, Fang Zhou, Junxia Yu, Ruan Chi

**Affiliations:** 1 Hubei Three Gorges Laboratory, Yichang 443007, China; q541702060@163.com (K.W.); yanling95@163.com (Y.C.); rac@wit.edu.cn (R.C.); 2 Key Laboratory for Green Chemical Process of Ministry of Education, Engineering Research Center of Phosphorus Resources Development and Utilization of Ministry of Education, Wuhan Institute of Technology, Wuhan 430205, China; jinyongtong723@163.com (Y.J.); fzhou@wit.edu.cn (F.Z.); yujunxia_1979@163.com (J.Y.)

**Keywords:** antibiotic resistome gene, metal resistome gene, compost, phosphate tailings, microbial community

## Abstract

Phosphate tailings are usually rich in phosphorus and some other mineral nutrients, which is very suitable for composting. In this study, 60 days of composting using phosphate tailings, chicken manure, and straw resulted in a significant decrease in total nitrogen (TN) content from 1.75 ± 0.12 g/kg to 0.98 ± 0.23 g/kg (*p* < 0.01), with a nitrogen retention of 56%, an increase in water-soluble phosphorus (Ws-P) from 3.24 ± 0.14 mg/kg to 7.21 ± 0.09 mg/kg, and an increase in immediate potassium (AK) from 0.56 ± 0.21 mg/kg to 1.90 ± 0.11 mg/kg (*p* < 0.05). Metagenomic sequencing showed little changes in the diversity and abundance of microbial communities before and after composting, but changes in species composition and the abundance of archaea, bacteria, and fungi resulted in differences in community structure before and after composting. Composting contributed to a lower gene abundance of ARGs and MRGs. The addition of phosphate tailings combined the functions of chemical regulation and nutrient enrichment, and its synergistic effect significantly optimized the nutrient cycling in the composting system.

## 1. Introduction

Phosphorus is an essential nutrient element for plant growth and development, playing an irreplaceable role in key biological processes such as plant physiological metabolism regulation, tissue structure formation, and stress resistance mechanisms [1]. Although the global soil total phosphorus content generally ranges between 500 and 2000 mg/kg, plant-available phosphorus accounts for only 1–5% of the total. Research indicates that approximately 57 million hectares of arable land worldwide suffers from insufficient available phosphorus supply, failing to meet the production requirements of major food crops. This phosphorus availability limitation has emerged as a critical challenge to global food security [2]. The application of phosphate fertilizers represents an effective strategy to enhance soil phosphorus availability and stimulate crop yields. Phosphate rock, as the primary raw material for fertilizer production, has an annual global mining output of approximately 2.2 billion tons, with about 85% consumed by the phosphate fertilizer industry [3,4]. Notably, with continued global population growth, current phosphate rock reserves are predicted to face depletion risks within the next 50–100 years [5]. This impending scarcity not only threatens the sustainability of phosphate fertilizer supply chains but also poses severe challenges to future global food security systems.

Phosphate tailings constitute the primary industrial waste generated during phosphate ore flotation processes. Due to limitations in ore grade and extraction technologies, approximately 30–40% of phosphate ore ultimately ends up in tailings, resulting in massive global accumulation of phosphate tailings annually [6,7]. The substantial stockpiling of phosphate tailings not only occupies extensive land resources but also poses significant environmental risks through residual components such as phosphate compounds and heavy metals. These contaminants contribute to soil degradation, vegetation destruction, and potential threats to human health [7,8]. On the other hand, the transfer of phosphorus from phosphate tailings to the surrounding soil and rivers also causes a huge waste of phosphorus resources [8]. Therefore, the resource utilization of phosphate tailings represents not only a crucial pathway toward sustainable development in the phosphate industry but also a vital strategy for ensuring global food security.

On the other hand, with the development of the agricultural economy, the production of agricultural solid waste (e.g., livestock and poultry manure and crop residues) is increasing year by year [9]. Composting is capable of converting organic matter (OM) from agricultural solid waste that can be converted into humus-rich fertilizers and is considered to be an effective and sustainable method for treating large quantities of agricultural solid waste. However, the slow decomposition process of organic matter in the composting system leads to the low humus content of the final fertilizer and long compost maturation period [10]. Additive incorporation enhances composting efficiency. Biochar accelerates OM transformation while mitigating heavy metal bioavailability and greenhouse emissions [11].

Many mineral additives, including diatomaceous earth, bentonite, and zeolite, can shorten the composting cycle and passivate pollutants such as heavy metals, antibiotics, etc. [10,12,13]. The mineral composition of phosphate tailings mainly includes dolomite (CaMg(CO_3_)_2_) and fluorapatite (Ca_5_(PO_4_)_3_F). Phosphate generated from the dissolution of phosphate tailings can reduce the pH of the system, alleviate the composting process produced by the conversion of NH_4_^+^ into NH_3_ to reduce the nitrogen loss in the process of composting, and increase the phosphorus content [14,15]. It is worth noting that heavy metals such as Cu and As associated with phosphate tailings and antibiotics carried by livestock manure may pose a risk to the environment when applied to the soil. However, little is currently known about the changes in the abundance of heavy metal resistance genes (MRGs) and antibiotic resistance genes (ARGs) during composting of phosphate tailings and livestock manure [7,16].

Therefore, this study conducted composting experiments using phosphate tailings and livestock manure, investigating the dynamic variations of MRGs, ARGs, and microbial community interactions through metagenomic sequencing. The findings aim to provide scientific insights for enhancing the resource utilization of phosphate tailings and improving the biosafety of tailings-derived fertilizers.

## 2. Material and Methods

### 2.1. Composting and Sampling

Chicken manure is high in nitrogen and carries functional microorganisms such as thermophilic bacteria, which can effectively accelerate the decomposition of substances. So, it was mixed with straw and phosphorus tailings powder for composting. Fresh chicken manure (CM) and straw (RS) were obtained from a farm outside Wuhan, Hubei, China, and phosphorus tailings were from Xingfa Chemical Group in Yichang, Hubei, China. Before composting, the chicken manure was air-dried outdoors for 5 days until the quality remained unchanged, straw was cut into 3–5 cm pieces, and phosphorus tailings were crushed and sieved through a 10-mesh screen. To speed up decomposition, raise compost temperature, and promote phosphorus tailings dissolution, phosphorus tailings powder, straw powder, and animal manure were mixed in a 1:2:3 weight ratio, with a total weight of 5 kg and moisture content adjusted to 50–60%. The composting device was a self-built insulated foam box that was 550 mm × 400 mm × 250 mm in size. The composting lasted 60 days, with turning every 3 days in the first 3 weeks and weekly afterward, starting from day 3.

A 20 g sample was collected from a random area at a depth of 25 cm on days 0 and 60 of composting, respectively, using three parallel samples each time, and each sample was divided into two parts for physicochemical index analysis and macro-genome sequencing to explore the changes in each index before and after composting. Group A represents the before composting, and group B represents the after composting.

### 2.2. Determination of Physicochemical Indexes

The water-soluble phosphorus (Ws-P) content of the samples was determined by extraction with deionized water and the colorimetric method using ammonium vanadium molybdate [17], and the total nitrogen (TN) content was determined by a nitrogen tester (KDN-2C, Shanghai Qianshi Instrument Co., Ltd., Shanghai, China). The compost samples were degraded in a digestion oven consisting of potassium sulphate–copper sulphate in an extracting solution at 375 °C before the determination (Sharifi et al., 2009, [18]), and AK was extracted with 1 mol/L nitric acid and 2% (m/V) citric acid solution and determined by the flame photometric method [19].

### 2.3. Metagenome Analysis

After collecting the compost samples, 1.0 g of compost samples was subjected to DNA extraction using the E.Z.N.A.^®^ Soil DNA Kit (Omega Bio-tek, Norcross, GA, USA), and the extracted DNA was used by Shanghai Meiji Bio-medical Science and Technology Co., Ltd. (Shanghai, China) to construct a PE library through Covaris M220 (Genetics, Shanghai, China) and to perform macro-genome sequencing (Shanghai Majorbio Bio-Pharm Technology Co., Ltd., Shanghai, China) using Illumina. The PE library was constructed by Covaris M220 (Genetics, Shanghai, China), and macro-genome sequencing was performed using the Illumina NovaSeq (Illumina, San Diego, CA, USA) sequencing platform (Shanghai Majorbio Bio-Pharm Technology Co., Ltd., Shanghai, China).

Gene sequences were clustered using CD-HIT (version 4.6.1) to construct non-redundant gene sets [20]. The amino acid sequences of the non-redundant gene set were compared with the NR, KEGG, and CARD databases using Diamond (version 0.8.35), respectively (the BLASTP (version 2.14.0) comparison parameter was set with an expectation value of 1 × 10^−5^), to obtain information on the abundance of species in the samples, the abundance table of KEGG functional genes, and the abundance of ARGs [21,22].

### 2.4. Statistical Analysis

The experimental data were expressed as mean ± standard deviation (mean ± SD). Statistical analyses were performed using R software (version 4.2.0), with the “car” package for analysis of variance (ANOVA), the significance level of differences between groups set at *p* < 0.05, and the “agricolae” package for analysis of multiple comparisons and the labeling of significance letters. The built-in Rstudio function “fisher.test ()”was used for Fisher’s exact test. A heat map was drawn using the pheatmap package to characterize the changes in the abundance of key metabolic genes before and after composting. The Mantel test based on Bray–Curtis distance matrix was analyzed and visualized by “linkET” and “ggplot2” packages to reveal the correlation between microorganisms and key metabolic genes as well as the correlation between genes. Origin Pro 2021 software was used to generate stacked histograms to characterize the changes in microbial species and their abundance before and after composting.

## 3. Results and Discussion

### 3.1. Changes in Physicochemical Properties of Compost

Significant changes in the physico-chemical properties of the composting system were observed after a 60-day fermentation cycle. As shown in Table 1, the pH of the system increased continuously from the initial 7.32 ± 0.15 to 8.68 ± 0.21 at the end of fermentation (*p* < 0.05), showing a typical alkalinization trend. This alkalizing trend may be due to the accumulation of alkaline substances produced by the ammonification of nitrogenous substances due to microbial fermentation in the system that promotes the decomposition of organic matter, thus affecting the pH of the system [23]. The dynamic monitoring of nutrient elements showed that the total nitrogen (TN) content decreased significantly from 1.75 ± 0.12 g/kg to 0.98 ± 0.23 g/kg (*p* < 0.01), and the nitrogen retention reached 56%, which was higher than that of the traditional composting process with about 50% or so nitrogen retention [24]. The water-soluble phosphorus (Ws-P) content was significantly enhanced from 3.24 ± 0.14 mg/kg to 7.21 ± 0.09 mg/kg (*p* < 0.01), and the fast-acting potassium (AK) content increased simultaneously from 0.56 ± 0.21 mg/kg to 1.90 ± 0.11 mg/kg (*p* < 0.05).

Microorganisms in the composting system can convert insoluble phosphorus from phosphate tailings into soluble phosphorus by secreting organic acids. In addition, organic acids also help to reduce the pH of the system, and the hydrolysis of NH_4_^+^ is inhibited under acidic conditions, preventing the conversion of NH_4_^+^ to NH_3_ and alleviating nitrogen loss [14,23,24]. Therefore, the incorporation of phosphate tailings was not only effective as a chemical additive to reduce nitrogen losses involved in nutrient cycling but also increased the soluble phosphorus content in the composting system. This result suggests that the co-fermentation system of phosphate tailings and chicken manure straw can effectively promote the conversion and retention of nutrients in the composting process.

### 3.2. Microbial Diversity and Community Succession

The alpha diversity index analysis showed (Table 2) that Good’s coverage indices were all above 0.94, indicating that the sequencing depth was sufficient to detect most of the microbial samples in the composting system. Bacteria had the highest diversity index and richness index compared to archaea and fungi, indicating that bacteria were the dominant microorganisms in the composting system. After 60 days of composting, the Shannon diversity index of the bacterial community increased from 5.43 ± 0.05 to 5.48 ± 0.01 (*p* > 0.05), and the bacterial community consistently maintained a high level of diversity despite a decrease in the ACE richness index from 3906.66 ± 23.31 to 3827 ± 34.36. The Shannon index and ACE index of the fungal community decreased from 5.59 ± 0.01 and 985.21 ± 14.61 to 5.45 ± 0.09 and 960.43 ± 33.09, respectively, whereas the Shannon index of the archaeal community decreased from 3.49 ± 0.09 to 3.42 ± 0.29 and the ACE index increased from 163.48 ± 2.59 to 164.63 ± 5.10. Although the changes in the diversity and abundance of archaea, bacteria, and fungi were not significant, composting had a tangible impact on the composition of the microbial community, with changes in species abundance in response to selective environmental pressures and inter-species competition.

**Figure 1:**
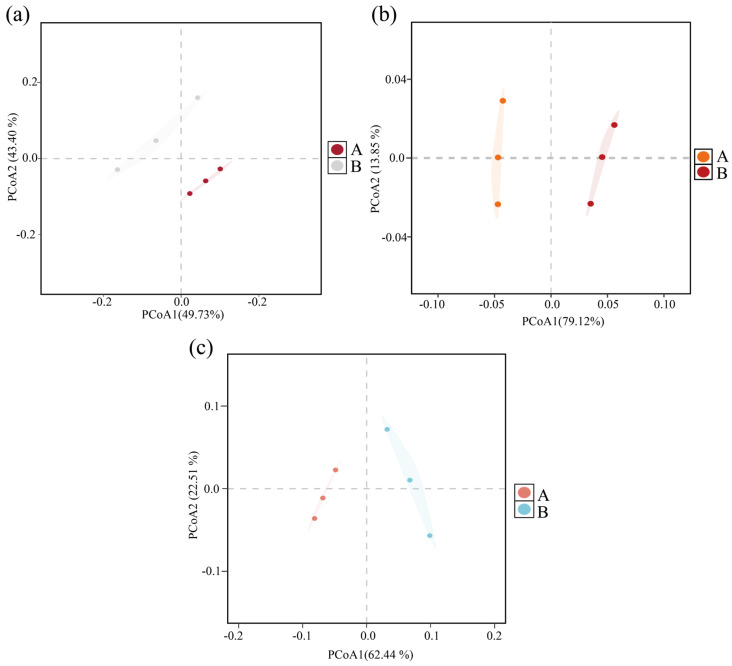
Bray–Curtis PCoA analysis based on differences in community structure at the microbial genus level before and after composting. (**a**) Archaea; (**b**) bacteria; (**c**) fungi. (A: before composting; B: after composting).

The community structure variability of archaea, bacteria, and fungi before and after composting was analyzed by principal coordinate analysis (PCoA) based on the Bray–Curtis distance matrix. As shown in Figure 1, the PCoA results explained 93.13% of the variability of the archaeal community (Figure 1a), 92.97% of the bacterial community (Figure 1b), and 84.95% of the fungal community (Figure 1c), indicating that both the bacterial and fungal communities were highly significant overall.

**Figure 2:**
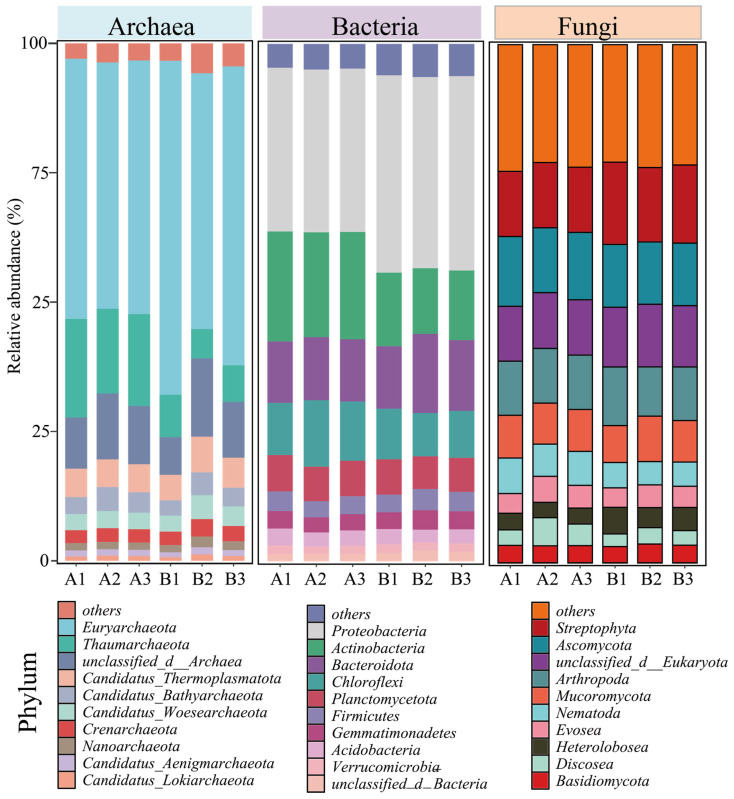
Changes in relative abundance of archaea, bacteria, and fungi at the phylum level prior to and subsequent to composting. (A: before composting; B: after composting).

Although the diversity and abundance of the archaeal community did not change significantly, the relative abundance of Euryarchaeota in the archaeal community increased from 48.94 ± 1.09% before composting (A) to 57.38 ± 6.30% after the composting (B) Figure 2. The Euryarchaeota phylum includes methanogens that drive methane production, *Bacillus salinarius*, and some thermophilic aerobes and anaerobes, which are common dominant bacteria in composting systems [25]. The increase in Euryarchaeota abundance suggests that the composting process carries out organic matter degradation. Chicken manure often contains high levels of nitrogen as well as organic matter, and during the composting process, the emission of malodorous gases (CH_4_, NH_3_) from the decomposition of organic matter is a critical and unavoidable problem, which also implies that nitrogen loss occurs in the system [26]. Thaumarchaeota is widely present in various environments, including soils, oceans, and freshwaters. As a chemoenergetic autotrophic microorganism, it can use ammonia as an energy source to participate in the nitrogen cycling process and optimize the nutrient structure of fertilizers by regulating nitrification in the system to reduce N_2_O emissions [27], but the abundance of the Thaumarchaeota phylum has also been reduced from 17.73 ± 1.08% to 6.98 ± 1.02% before composting because of unavoidable nitrogen loss.

The change in the abundance of the bacterial community was particularly significant, with the Proteobacteria and Actinobacteria phyla being the two most abundant phyla in the composting system, and with the abundance of Proteobacteria increasing from 31.56 ± 0.08% before composting to 37.54 ± 0.49% after composting. However, the abundance of Actinobacteria decreased from 20.75 ± 0.40% before composting to 13.47 ± 0.62% after composting (Figure 2). Both the Proteobacteria and Actinobacteria phyla are capable of secreting laccase and β-glucosidase, which enable them to rapidly degrade lignocellulose. Proteobacteria can also convert some soluble sugars into monosaccharides and short-chain fatty acids. Actinobacteria, by secreting a variety of antibiotics, can kill pathogenic microorganisms. Therefore, they become dominant microorganisms in the composting system [28,29]. However, the abundance of Actinobacteria decreases after composting, which may be due to interspecies competition.

Ascomycota, as the largest and most diverse phylum of fungi, contains saprophytes (e.g., *Aspergillus*, *Penicillium*) that play key roles in the composting process, especially in the degradation of recalcitrant organic matter, microbial interactions, and the regulation of the compost quality. Therefore, the abundance of Ascomycota was always maintained above 12% before and after composting, and it became the dominant fungus in the system (Figure 2) [30].

### 3.3. Metabolic Function Analysis

**Figure 3:**
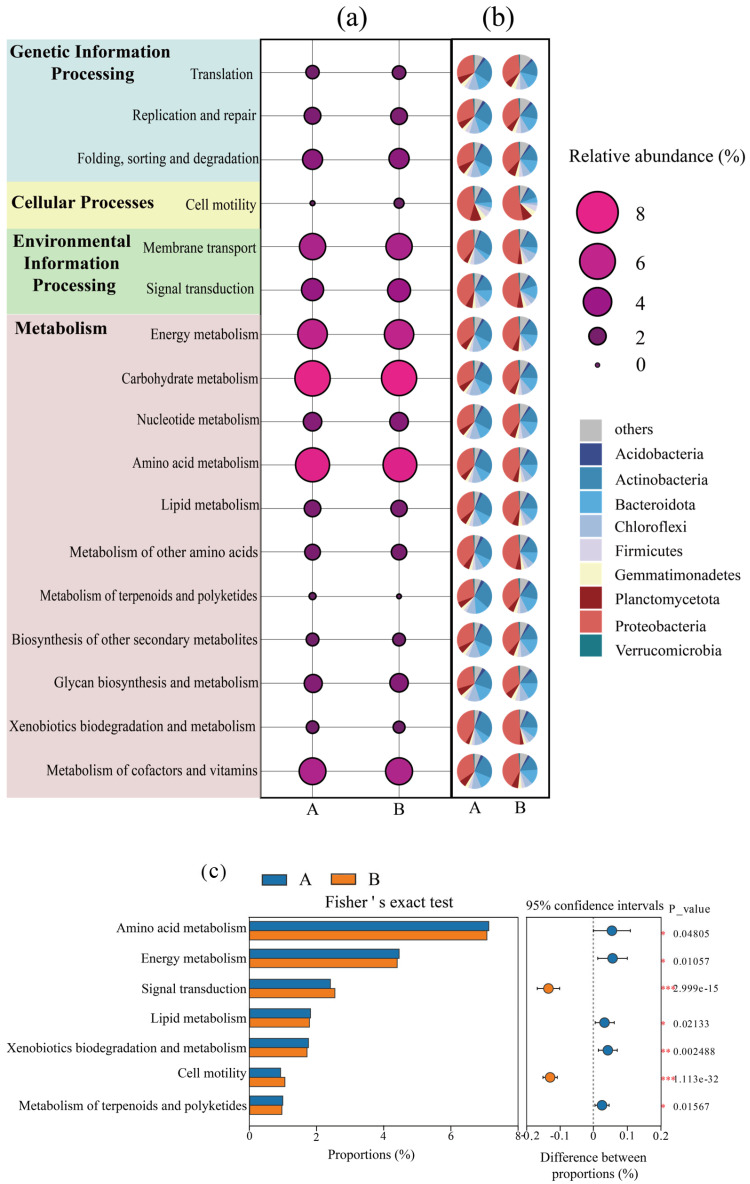
Changes in relative abundance of microbial metabolic pathways before and after composting; (**a**) changes in relative abundance of the KEGG secondary metabolic pathway in the composting system; (**b**) contribution of different species to metabolic pathways; (**c**) Fisher’s exact test for KEGG secondary metabolic pathways. (A: before composting; B: after composting. *, *p* < 0.05; **, *p* < 0.01; ***, *p* < 0.001).

In order to investigate the changes in the overall metabolic functions of microorganisms in the system before and after composting, a functional enrichment analysis was performed based on the KEGG database. Figure 3a shows that the relative abundance of pathways involving microbial “membrane transport”, “signal transduction”, “amino acid metabolism”, “carbohydrate metabolism”, “energy metabolism”, and “metabolism of cofactor and vitamin” in the composting system was high, indicating that microorganisms were able to efficiently decompose the materials in the composting system and perform their own metabolism and proliferation [31]. Figure 3b shows that the Proteobacteria and Actinobacteria phyla contribute significantly to almost all metabolic pathways, indicating that bacteria from these two phyla are the main bacteria dominating these metabolic pathways, which also corresponds to the abundance of the Proteobacteria phylum and Actinobacteria in Figure 2.

The metabolic pathways with significant changes in abundance before and after composting were explored by Fisher’s exact test (Figure 3c), in which the abundance of amino acid metabolism, energy metabolism, lipid metabolism, biodegradation and metabolism of exogenous organisms, and metabolism of terpenes and polyketides were significantly reduced after composting was completed (*p* < 0.05). Amino acid metabolism is the main pathway for providing energy to microorganisms during compost decomposition, which may be due to the decrease in the abundance of amino acid metabolism due to the reduction of readily degradable carbon sources in the later stages of composting [32,33]. Thus, the abundance of energy metabolism, lipid metabolism, biodegradation and metabolism of exogenous organisms, and metabolism of terpenoids and polyketides were significantly reduced after composting (*p* < 0.05). Notably, the abundance of signaling (e.g., group sensing, two-component system) and cell motility (e.g., flagellar assembly, chemotaxis) pathways, which are closely related to microbial environmental adaptations, were significantly increased in the latter part of the composting section (*p* < 0.001), indicating that the composting process enhanced microbial energetic inter-cellular communication and group migration, which may also be a way of shaped microbes to synergistically cope with environmental stresses [34].

**Figure 4:**
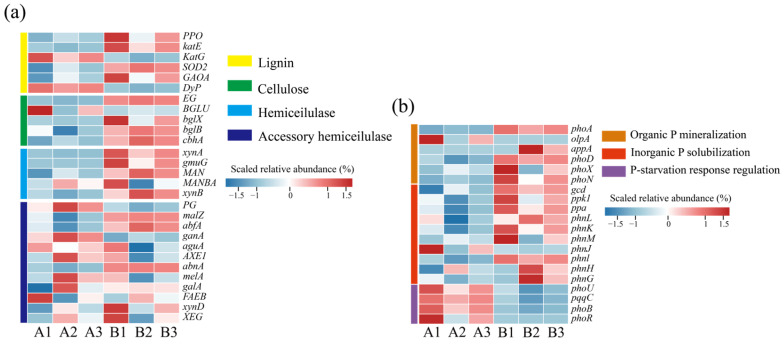
Changes in abundance of microbial functional genes at different composting stages. (**a**) Lignin and cellulose degradation genes; (**b**) phosphorus solubilization functional genes. (A: before composting; B: after composting).

The degradation of lignin and cellulose is a key process in humus formation. Based on the functional annotation of genes in the KEGG database, this study systematically analyzed the dynamic changes in genes related to the metabolism of lignin and cellulose in the composting process. Figure 4a shows the changes in the abundance of genes related to lignin and cellulose degradation before and after composting. Among the genes related to lignin degradation, the genes encoding catalase (*katG*, *katE*), peroxidase (*DyP*), superoxide dismutase (*SOD2*), and coenzymes (*GAOA*, *PPO*) were significantly up-regulated at the later stage of composting, and these oxidative enzyme systems play important roles in the breaking of the aromatic ring and free radical scavenging of lignin [35]. Meanwhile, cellulose degradation-related genes showed a trend of simultaneous activation, including endoglucanase (*EG*), cellulose 1,4-β-cellobiose glycosylase (*cbhA*), and β-glucosidase (*BGLU*, *bglX*, *bglB*), and the up-regulation of the abundance of these genes was closely associated with the process of dissociation of the crystal structure of cellulose and hydrolysis of cellobiose. Further analysis revealed that the hemicellulase gene cluster (*xynA*, *xynB*, *MANBA*, *abfA*, *GLA*, *gala*, *melA*, *ganA*, *malZ*) showed a similar dynamic pattern of change with cellulose degradation genes, indicating that there is a synergistic effect between the two in the process of straw degradation and that hemicellulase can decompose xylan, mannan, and other matrices by unraveling their encapsulation on cellulose microfilaments and lifting their wrapping effect on cellulose microfilaments, thus enhancing the effect of cellulase and ultimately realizing the degradation of cellulose through the cascade reaction of multienzyme [35,36].

Microorganisms play an important role in the dissolution of phosphate tailings [37]. Phosphorus metabolism-related functional genes showed significant stage characteristics during the composting process, and it is noteworthy that the phosphorus starvation-responsive regulatory gene clusters (*phoU*, *pqqC*, *phoB*, and *phoR*) showed significantly high expression levels at the initial stage of composting [38]. This phenomenon may be attributed to the low bioavailability of organic phosphorus in chicken manure, while the inorganic phosphorus from phosphate tailings has not been sufficiently solubilized, resulting in the system being in a state of effective phosphorus deprivation, which makes the microorganisms highly enriched in genes of the phosphorus starvation stress-regulatory pathway. The abundance of phosphorus metabolism genes changed before and after composting. At the later stage of composting, the abundance of alkaline phosphatase genes (*phoA*, *phoD*, *phoX*), acid phosphatase genes (*olpA*, *alpA*), and genes coding for the C-P lyase multienzymatic complex (*phnG-H-J-M-K*) was elevated, suggesting that the microorganisms catalyzed the dephosphorylation of the organophosphorus components of the system, such as phytate and phospholipids, by secreting the phosphatase system phosphorylation to achieve the mineralized decomposition of organic phosphorus [39]. At the same time, the abundance of functional genes related to inorganic phosphorus activation (*ppa*, *ppk1*, *gcd*, *pqqC*) was synchronously up-regulated, and the microbial-mediated phosphate solubilization mechanism was activated to promote phosphate tailings dissolution [1]. This multigene synergistic expression pattern eventually led to a significant increase in the soluble phosphorus content of the heap and promoted the conversion of insoluble phosphorus to bio-effective phosphorus.

### 3.4. Changes in Resistance Genes

**Figure 5:**
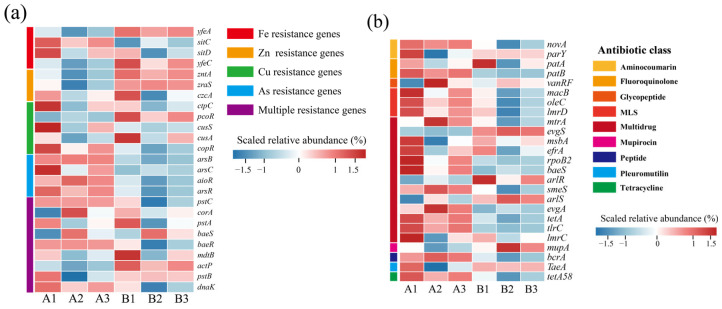
Changes in abundance of resistance genes at different composting stages. (**a**) Heavy metal resistance genes; (**b**) antibiotic resistance genes. (A: before composting; B: after composting).

Changes in the abundance of heavy MRGs during the mixed composting of phosphate tailings with chicken manure straw showed significant stage dynamics (Figure 5). The initial abundance of MRGs was higher overall during the initial stages of composting, which may have resulted from the drive of heavy metals (e.g., Cu, As) in phosphate tailings on the evolution of indigenous microbial communities (Figure 5a) [15,40]. As the composting process advances, microbial metabolic activity is enhanced, and lignin and cellulose are degraded, accompanied by the generation of a large number of extracellular polymers, such as humic acid, which, by chelating with heavy metal ions, change the morphology of the heavy metals and reduce the bio-efficacy of the heavy metals, thus decreasing the biotoxicity of the heavy metals, such as Cu, As, and so on [35,36]. At the same time, along with the decomposition of nitrogenous organic matter and NH_3_ production in the composting system, the pH of the system continued to increase, which promoted the precipitation of heavy metals with hydroxyl/carbonates in the system, further weakening the selective pressure of heavy metals on microorganisms [41,42]. Although there was an increase in the abundance of *cusA*, which expresses copper efflux system proteins; the *zutA* gene, which is a zinc-translocating ATPase; and the *yfeC* gene, which is an iron-translocating system-binding protein, at the end of composting. This ultimately resulted in an overall decreasing trend in the abundance of MRGs.

Antibiotics are added to livestock feeds in large quantities due to their function in promoting growth, weight gain, and health maintenance. However, about 58.3% of antibiotics cannot be absorbed by livestock or poultry and are excreted in livestock and poultry manure [43]. Therefore, the presence of ARGs in compost has potential ecological risks for fertilizer use. The top 25 ARGs in terms of abundance are listed in Figure 5b. Since chicken manure was not subjected to composting and fermentation treatment, ARGs such as fluoroquinolones (*parY*, *patA*) and aminocoumarins (*novA*) macrolides (*macB*, *oleC*, *lmrD*) were generally high in the early stages of composting. Notably, *vanRF*, *rpoB2, tlrC*, *imrC*, and *mupA* avoid antibiotic action by modifying the antibiotic action site, and the remaining genes, such as the tetracycline efflux pump gene, *tetA*, and macrolide antibiotic efflux pump genes, *oleC*, and *lmrD*, resist the effects of antibiotics by inducing antibiotic efflux. As a result of experiencing the effects of high temperatures generated by composting, a large number of pathogenic bacterial hosts were killed in the composting system at the end of composting, which contributed to the decrease in the abundance of ARGs [44], and the reduction of the toxic effects of heavy metals during composting also weakened the co-selective pressures of the heavy metals on the ARGs, which resulted in a decrease in the abundance of the ARGs [45].

**Figure 6:**
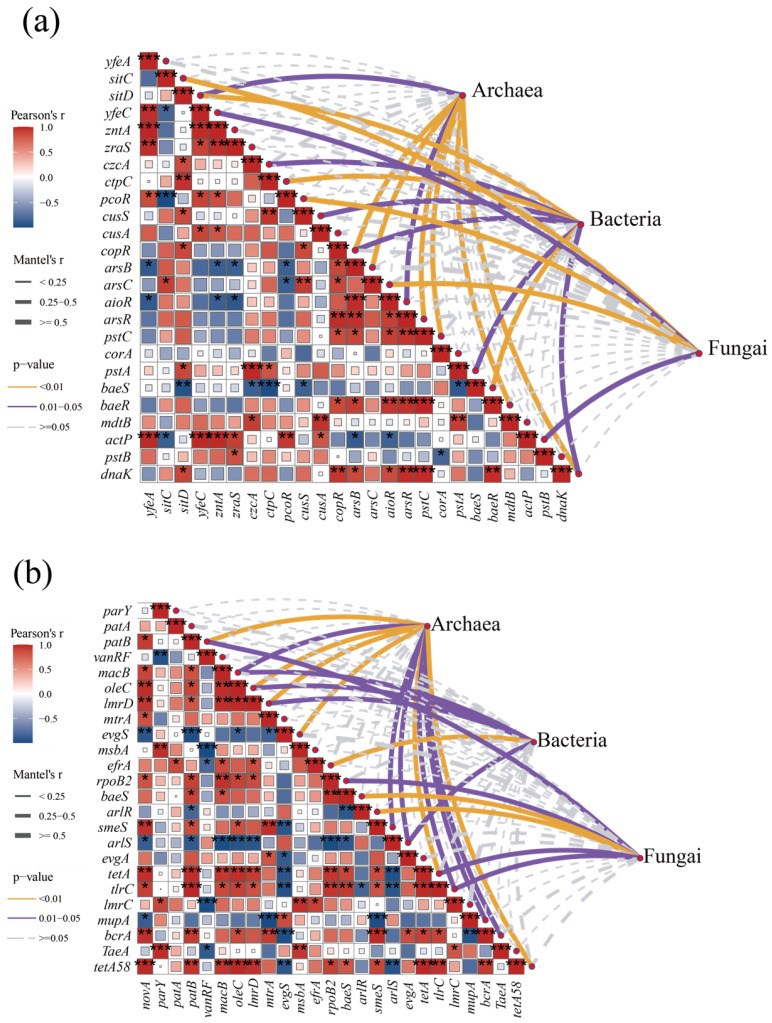
Correlation between resistance gene abundance and microbial abundance. (**a**) Correlation between heavy metal resistance genes and microorganisms; (**b**) correlation between antibiotic resistance genes and microorganisms. (*, *p* < 0.05; **, *p* < 0.01; ***, *p* < 0.001).

As heavy MRGs and antibiotic resistance gene carriers, changes in microbial abundance were significantly correlated with changes in resistance gene abundance [44]. Therefore, a correlation between the abundance of microbial communities and the abundance of MRG and ARGs in the composting process was investigated based on the analysis of Mantle’s test (Figure 6). The abundance of archaea was significantly and positively correlated (Mantel’s r ≥ 0.5, *p* < 0.05) with the abundance of arsenic resistance genes (*arsB*, *arsR*, *aioR*) (Figure 6a). *Methanosarcina acetivorans*, a member of the generalized archaeal phylum, has been reported to mediate the reduction and methylation of As(V) during methane biogenesis; thus, archaeal communities may metabolize arsenic in the environment more strongly than bacteria and fungi and, thus, show higher relevance [46]. As the predominant microbial community in composting systems, bacteria have a wide range of metabolic diversity and, therefore, a wide range of resistance to a variety of heavy metals such as iron, copper, zinc, and arsenic. The multi-metal resistance characteristics of bacteria reflect their ecological competitive advantages in complex composting environments. Fungi showed a lower correlation with heavy MRGs compared to archaea and bacteria, while significant associations were found with the abundance of iron/manganese transporter proteins (*sitC*), iron transporter proteins (*yefeC*), copper resistance regulatory proteins (*pcoR*), and arsenate reductase genes (*arsC*).

ARGs were more closely correlated with microbial abundance compared to heavy MRGs (Figure 6b), suggesting that antibiotics have a greater impact on microbial communities compared to heavy metals. Unlike heavy MRGs, archaea with lower microbial abundance were more closely associated with ARGs, higher than bacteria and fungi; thus, the diversity index of archaea in the later stages of composting was accompanied by a decrease in the abundance of ARGs. Same as prokaryotes, both archaea and bacteria showed a significant correlation with macrolide ARGs (*macB*, *oleC*, lmrD); in addition, archaea and bacteria also showed a significant positive correlation with multidrug resistance genes (*arlS*, *erfA*) as well as with tetracycline resistance genes (*tetA*), etc. (Mantel’s r ≥ 0.5, *p* < 0.05), whereas fungi mainly produced a significant correlation with broad-spectrum resistance genes such as *ropB2*, *baeS*, *arlR*, and *tlrC*, indicating that fungi have a broader multidrug resistance mechanism to antibiotics.

It is worth noting that both ARGs and heavy MRGs are also negatively correlated with each other within themselves. And in addition to environmental factors such as antibiotics or heavy metals driving the enrichment of more tolerant microorganisms through selective pressures, it may also be due to competition between microorganisms, leading to changes in the abundance of host microorganisms for resistance genes and interspecies interactions remodeling the distribution pattern of resistance genes, thus affecting the correlation between resistance genes.

## 4. Conclusions

After 60 days of co-composting phosphatic tailings with livestock manure and straw, the system showed significant nutrient-regulation effects. The total nitrogen retention rate reached 56%, with Ws-P and AK contents increasing significantly from 3.24 ± 0.14 mg/kg to 7.21 ± 0.09 mg/kg and from 0.56 ± 0.21 mg/kg to 1.90 ± 0.11 mg/kg, respectively. PCoA revealed that composting altered the structure of microbial communities. The abundance of Proteobacteria increased from 31.56 ± 0.08% pre-composting to 37.54 ± 0.49% post-composting, while that of Actinobacteria fell from 20.75 ± 0.40% to 13.47 ± 0.49%. During composting, amino acid metabolism and energy metabolism changed significantly, with shifts in the abundance of Proteobacteria and Actinobacteria driving changes in metabolic pathways. Post-composting, the abundance of genes related to carbohydrate metabolism and phosphorus metabolism increased while that of heavy metal and antibiotic resistance genes decreased overall. This indicates that microbial metabolic activities can enhance the agricultural safety and nutrient-release properties of compost products.

## Figures and Tables

**Table 1:** Changes in nutrient parameters (mean ± SD) of phosphate tailings fertilizer before and after composting.

Parameters	A	B
TN (g/kg)	1.75 ± 0.12 a	0.98 ± 0.23 b
Ws-P (mg/kg)	3.24 ± 0.14 b	7.21 ± 0.09 a
AK (mg/kg)	0.56 ± 0.21 b	1.90 ± 0.11 a
pH	7.32 ± 0.15 b	8.68 ± 0.21 a

Different letters indicate significant differences before and after composting. A: before composting; B: after composting.

**Table 2:** Analysis of microbial community alpha diversity indices (mean ± SD).

	Bacteria		Fungi		Archaea	
	A	B	A	B	A	B
Shannon	5.43 ± 0.05	5.48 ± 0.01	5.59 ± 0.01	5.45 ± 0.09	3.49 ± 0.09	3.42 ± 0.29
ACE	3906.66 ± 23.31	3827 ± 34.36	985.21 ± 14.61	960.43 ± 33.99	163.48 ± 2.59	164.63 ± 5.10
Good’s coverage	0.998 ± 0.001	0.997 ± 0.001	0.944 ± 0.004	0.945 ± 0.001	0.989 ± 0.001	0.984 ± 0.003

A: before composting; B: after composting.

## Data Availability

The original contributions presented in this study are included in the article. Further inquiries can be directed to the corresponding author.
